# Transforming youth mental health services in a large urban centre: ACCESS Open Minds Edmonton

**DOI:** 10.1111/eip.12813

**Published:** 2019-06-27

**Authors:** Adam Abba‐Aji, Katherine Hay, Jill Kelland, Christine Mummery, Liana Urichuk, Cindy Gerdes, Mark Snaterse, Pierre Chue, Shalini Lal, Ridha Joober, Patricia Boksa, Ashok Malla, Srividya N. Iyer, Jai L. Shah

**Affiliations:** ^1^ Alberta Health Services Edmonton Zone Edmonton Alberta Canada; ^2^ Department of Psychiatry University of Alberta Edmonton Alberta Canada; ^3^ ACCESS Open Minds Edmonton Edmonton Alberta Canada; ^4^ ACCESS Open Minds (Pan‐Canadian Youth Mental Health Services Research Network) Douglas Mental Health University Institute Montreal Quebec Canada; ^5^ School of Rehabilitation, Faculty of Medicine l'Université de Montréal Montreal Quebec Canada; ^6^ Department of Psychiatry McGill University Montreal Quebec Canada; ^7^ Centre de recherche du Centre hospitalier de l'Université de Montréal (CRCHUM) Montreal Quebec Canada; ^8^ Prevention and Early Intervention Program for Psychosis (PEPP) Douglas Mental Health University Institute Montreal Quebec Canada

**Keywords:** access, case identification, service transformation, youth mental health, Canada

## Abstract

**Aim:**

This paper outlines the transformation of youth mental health services in Edmonton, Alberta, a large city in Western Canada. We describe the processes and challenges involved in restructuring how services and care are delivered to youth (11‐25 years old) with mental health needs based on the objectives of the pan‐Canadian ACCESS Open Minds network.

**Methods:**

We provide a narrative review of how youth mental health services have developed since our engagement with the ACCESS Open Minds initiative, based on its five central objectives of early identification, rapid access, appropriate care, continuity of care, and youth and family engagement.

**Results:**

Building on an initial community mapping exercise, a service network has been developed; teams that were previously age‐oriented have been integrated together to seamlessly cover the age 11 to 25 range; early identification has thus far focused on high‐school populations; and an actual drop‐in space facilitates rapid access and linkages to appropriate care within the 30‐day benchmark.

**Conclusions:**

Initial aspects of the transformation have relied on restructuring and partnerships that have generated early successes. However, further transformation over the longer term will depend on data demonstrating how this has impacted clinical outcomes and service utilization. Ultimately, sustainability in a large urban centre will likely involve scaling up to a network of similar services to cover the entire population of the city.

## 1 INTRODUCTION

The availability of effective services to adolescents and young adults facing mental health and addiction challenges has long been insufficient (Malla et al., 2018). Encouragingly, youth‐friendly interventions at early stages of need offer long‐term benefits for outcomes (Clark & Unruh, 2009). It is in this context that the ACCESS Open Minds (ACCESS OM) initiative has facilitated service transformation around five common objectives in different contexts across Canada (Malla et al., 2018). Here, we describe how efforts towards these objectives have manifested in service and system transformation in a large urban centre in Edmonton, Alberta. The city has a population of 1.32 million, 31% of which is under the age of 25 along with the second largest urban Indigenous population in Canada and a large homeless population (estimated at 1700), mostly young adults (Statistics Canada, 2016). Edmonton's city centre, where its ACCESS OM program is located, has a population of 17 000 youth aged 10‐24 and a relatively high level of unemployment, with one‐third of individuals having no income (Statistics Canada, 2016). Thus, the Edmonton ACCESS OM program serves a local population with complex needs, amidst a young, diverse and growing city.

Mental health services in Alberta are primarily provided by Alberta Health Services (AHS) and organized according to geographic zones, one of which is Edmonton and its surrounding regions. Prior to joining ACCESS OM, the Edmonton Zone Addiction and Mental Health program had historically provided a continuum of specialized care options for youth experiencing mental health and/or substance use concerns (Table 1). However, despite initial attempts at integration (e.g., the creation of a young adult portfolio specifically for youth aged 16‐25), this age group remained at risk for poor outcomes due to the system's age‐bounded service delivery model, the lack of engagement in effective and appropriate care options and a disorganized or poorly supported transition processes (Alberta Health Services, 2017). Rapid access to care was limited except for those who met criteria for early psychosis, services were fragmented with little continuity across teams, and there was no active follow‐up for acute patients who did not meet specific Diagnostic and Statistical Manual (DSM) diagnostic criteria. Consequently, service providers were likely to miss opportunities for early identification and intervention.

**Table 1:** Alberta Health Services Edmonton Zone—developmentally appropriate inpatient and community outpatient programming as of 2014

Edmonton early psychosis intervention clinic	For individuals experiencing a first episode of psychosis
Transitional youth services	Designed to assist individuals with multi‐system needs and co‐occurring difficulties graduating from child to adult services
Challenge by choice	Providing social and recreational programming options for those under the age of 30
Young adult treatment team	For young adults accessing addiction services, with a 90‐day residential program
Young Adult Assessment, Treatment, and Reintegration Unit at Alberta Hospital Edmonton	Offering acute care admissions in a youth‐friendly environment with a 90‐day follow‐up post discharge
Eating Disorders Program	Operated out of the University of Alberta Hospital with a continuum of inpatient, intensive day treatment and outpatient services

These service gaps became an important reason for partnership when the Edmonton Zone was approached to join the ACCESS OM initiative. Building on this, the Edmonton Young Adult Addiction and Mental Health Services team organized a multidisciplinary ACCESS OM steering committee with representation across child, youth and family services, young adult and cross level (recovery through employment, education, housing and peer support) services, research and evaluation and decision support and analytics that highlighted areas of the service delivery system that ACCESS OM Edmonton should focus on.

## 2 COMMUNITY MAPPING

Prior to joining ACCESS OM, community mental health services for youth in Edmonton were sharply divided based on age. Children below the age of 18 attended child and adolescent mental health services (CAMHS), while those between 18 and 65 years of age attended general adult psychiatry. Although transfer of care from CAMHS to general adult services was typically initiated by the time the youth was 18 years old, this model lacked adequate continuity of care: 80% of youth (aged 16‐25) discontinued seeing their primary service provider in adult services after three visits and without a planned discharge (Alberta Health Services, 2018). Furthermore, despite utilizing high‐intensity services at high rates (with 30% of all psychiatric intensive care admissions being for those under 25 years old), individuals least likely to engage in appropriate care were those who were close to aging out of the service at their first contact (e.g., 17 years old); or who did not have adequate supports in navigating the access point (e.g., homeless youth, individuals who were unable to ask for help from their parents or guardians, or who had parents with mental health and substance use issues) (Alberta Health Services, 2018).

Given this noted gap, a young adult mental health portfolio had been established in 2014 with responsibility for strengthening mental health and addiction services for transition‐aged youth, quality improvement to reduce barriers to accessing mental health services, and collaboration in order to better meet the needs of youth and their families. In 2017, the Edmonton Young Adult Addiction and Mental Health Services team formally joined the national ACCESS OM project with the explicit goal of better meeting the needs of youth aged 11‐25. This directly led to the alignment of services between CAMHS and general adult psychiatry. The multidisciplinary steering committee then performed community mapping to identify service gaps and resource distribution within the context of socioeconomic demographic and population density needs.

This mapping also led to the development of community mental health services located along public transit routes within Edmonton. In May 2017, Edmonton opened its ACCESS OM site, a centrally located program to serve all help‐seeking youth aged 11‐25, but focused on engaging underserved and marginalized youth. Its goal was to provide a continuum of addiction and mental health services that would also act as a more accessible front door to youth seeking mental health services. Based on previous community mapping, the ACCESS OM clinic was established in the downtown Young Men's Christian Association (YMCA) building, easily accessible via public transportation and at no cost to service users, with a staffing model developed to effectively engage and assess those presenting with various addiction and mental health issues.

## 3 ACCESS CLINICIANS

One of the central transformations funded through ACCESS OM is the creation of ACCESS Clinician positions. As with ACCESS Clinician positions at other ACCESS OM sites, these are full‐time individuals (mental health professionals with a related post‐secondary degree), who initially engage and then maintain a consistent connection to an otherwise complex system for young people and their families. Through mobile, barrier‐free availability in community settings, three ACCESS Clinicians meet young people and their families in their homes, community agencies, schools, coffee shops and work places. This flexibility enables them to build a therapeutic, trusting, and non‐judgmental relationship with a youth or young adult and their family and carers, and then (if needed) to leverage that relationship to encourage and support clinical connections to a health care professional.

## 4 EARLY IDENTIFICATION

Efforts to improve early identification of youth in need of mental health services have focused on relationship‐building and simplifying pathways of care for initial presentations. This has been accomplished through direct and indirect public awareness campaigns by ACCESS OM, ranging from formal presentations in venues throughout the city to posting fliers in emergency rooms, suburban community health centres and primary care networks across Edmonton. Both modalities have outlined common presenting problems and available resources for youth experiencing mental health difficulties, as well as information for contacting the ACCESS Clinicians.

A significant investment has also been made in the ACCESS Clinician role and developing skills in motivational interviewing, behavioural activation, harm reduction approaches and strengths‐based models of care. To support successful implementation of this role, the AHS clinical informatics team was able to create an “Engagement” visit code to accurately account for the time spent and complex work required by ACCESS Clinicians to engage young people in care: the process of developing a trusting relationship and therapeutic alliance with a young person, even before traditional clinical interventions are provided.

ACCESS Clinicians are also regular attendees at agencies serving youth throughout the Edmonton area, many of which have established drop‐in spaces as a means to engage vulnerable groups. Prior to ACCESS OM, these agencies could identify young people who were experiencing mental health difficulties, but had few ways to facilitate access to appropriate mental health evaluations and services. The newfound relationship with ACCESS Clinicians provides agencies with a clear pathway to accessing services—without needing to wait until a threshold for emergency or urgent care is met. Thus, individuals, families and caregivers are now seen based on need rather than whether they meet a minimum threshold or stage of illness.

## 5 RAPID ACCESS

In addition to the mobile and flexible availability of ACCESS Clinicians, another facilitator of rapid access to care has been the development and opening of a physical ACCESS OM site. Prior to joining ACCESS OM, there was no “walk‐in clinic” for young adults with mental health needs in this large urbal centre. The ability to provide services in a drop‐in format matches the urgency expressed by youth (age 11‐25) to address their needs (Clark & Unruh, 2009). With the help of a youth advisory group, a site at the local Young Men's Christian Association (YMCA) community services building (centrally located and in non‐stigmatizing surroundings) was selected to be the location of the walk‐in clinic, which is staffed with 2.0 FTE (full‐time equivalent) mental health therapists, 1.5 FTE peer support workers and 1.0 FTE reception/administrative support person. Young people participated in the design with special attention to creating a safe, welcoming and comfortable space.

Walk‐in appointments are now available with an average wait of less than 30 minutes (Alberta Health Services, 2018) to a skilled counsellor or a peer support worker. An initial visit then opens the door to engaging in a solution‐focused counselling session and/or initial intake conversation or a more informal or social visit. Screening tools embedded into the clinic's visit documentation can flag concerns and easily identify areas that may need to be addressed clinically and those that might signal moderate to severe issues, including both mental illness and substance use/misuse.

Beyond the physical space itself, care is readily available at the ACCESS OM clinic by a multidisciplinary team comprised of four psychiatrists, three mental health therapists with background in psychology, two social workers, five addiction counsellors, two peer support workers, two occupational and two recreational therapists and one supported employment specialist, one nurse and one family counsellor. This involves a change in structure and functioning, not just location: most staff have been reoriented from clinic‐based appointments (where the young person had to come to them to receive service), to mobile availability in the location of choice (coffee shops, homes, schools, public libraries, etc), and staffing hours are now extended into the early evening (to improve access to services outside of regular business, school and work hours for young people and their families). Furthermore, education, skills training and clinical supervision of staff are based on the principles of recovery‐oriented care, harm reduction and self‐determination.

## 6 APPROPRIATE CARE IN 30 DAYS

The transformation of pathways to subsequent appropriate care has been approached in steps, iteratively gathering data and information from young people and their families to inform changes which are then implemented. Although no comparison data are available, within six months of opening the ACCESS OM clinic, young people reported a goal related to mental health 55% of the time, such as “feeling better” or “coping with my anxiety,” followed by seeking assistance with employment and career (32%) and community life functioning (31%) (Alberta Health Services, 2018). This prompted further development in the model, including:

1. The role of staff at the ACCESS OM clinic has evolved to include single session counselling; peer support workers have begun to focus more on follow‐up and engagement activities.
2. Service providers were required to develop drop‐in programming that met goal areas identified by individuals accessing services (and their families): employment and education support, 1:1 addiction counselling, group interventions such as social groups, mindfulness, Cognitive Behavioral Therapy (CBT), distress tolerance, and family psychoeducation programs.
3. In addition to the ACCESS Clinicians, psychiatrists have increased their clinic hours to address the high volume of referrals and offered weekend clinics to address backlogs when there were surges of referrals.
4. Health professionals from the Inpatient Unit (occupational therapy, recreation therapy, addiction counsellor) began to work one day per week at the ACCESS OM clinic to build relationships with community staff, increase their understanding of outpatient services, and facilitate seamless transition for youth who experience an inpatient acute care stay.
5. New resources were allocated to the young adult service in the following months including 2.0 FTE addiction counsellors, 2.0 FTE mental health therapists and a 1.0 FTE Family Peer Support Worker.

Thus, the model of service integration informed by data has allowed multiple needs of youth to be progressively addressed in an iterative manner over time (Hetrick et al., 2017).

## 7 CONTINUITY OF CARE BEYOND AGE 18

The transformation of continuity of care across the age 18 threshold began prior to ACCESS OM, when two programs spanning these age clusters (under 18 and over 18) together developed a shared vision. Initially, however, individuals accessing services outside of the young adult programs were still required to be referred to an entirely separate adult service when “aging out” of child/adolescent care—creating a jarring and potentially disengaging transition point.

In joining ACCESS OM, the multidisciplinary steering committee recognized that designing a transformation to phase out the notion of “aging out” involved issues such as improved understanding of mature minor status, consent to care for individuals under the age of 18, historical legacies (such as distinctions between child/adolescent and adult psychiatry) that were not designed with youth in mind, and understanding the intricacies of working with families during (eventual) transition periods. Now, due to ACCESS OM, youth aged 16‐18 continue to receive continuous services from the same young adult team for as long as is clinically necessary (until the age of 25). While it is relatively rare for individuals under the age of 14 to seek services at the ACCESS OM clinic, clear pathways to well‐developed child and adolescent services are in place after an initial assessment has been conducted. Following assessment in the ACCESS OM clinic, some youth are referred to specialized services such as Edmonton Early Psychosis Intervention Center or the Eating Disorders Program.

## 8 YOUTH AND FAMILY ENGAGEMENT

Prior to ACCESS OM, youth and family engagement was done through a third party such as Youth Empowerment Support Services and Canadian Mental Health Association. Now, the ACCESS OM clinic has itself taken on responsibility for directly engaging with youth and families. Youth and family advisors have been a part of the ACCESS OM Edmonton steering committee. They have been instrumental in developing welcome videos, promotional materials, assisting providers in their approach with young people and reviewing and providing feedback on strategic planning documents. Suggestion boxes and youth advisory meetings have been used to gather feedback and amplify the youth voice in our planning and implementation decisions. As part of the ACCESS OM project, staff have also developed and implemented an inclusive care guideline to formally address working with families and friends of young people (Table 2).

**Table 2:** Target audiences for the ACCESS OM Edmonton inclusive care guidelines

Individuals	To understand the importance of including family and friends in their recovery journey, and creative ways to keep these supports in place
Families	Education about the process of recovery and mental health and addiction, increasing understanding and skills to support their loved one and maintaining involvement during this transition phase
Staff	Understanding ways to speak to families and include them in care; all staff providing young adult services are now expected to self‐evaluate their inclusive care skills in their annual performance appraisal

A major catalyst for family engagement was the creation of a Family Peer Support Worker position, an AHS employee who is expected to practice from the viewpoint of lived experience as a parent/carer of a youth with substance misuse and mental illness in three main areas: assistance with system navigation, advocacy for the family voice with service providers, and support and understanding of the experience that youth and their families are going through. Family‐oriented groups led by this staff member have covered topics such as education about monitoring symptoms, medication side‐effects, learning coping strategies and improving communication.

## 9 THE UNIQUENESS OF ACCESS OM EDMONTON: COMMUNITY IMPACT

The advent of ACCESS OM has provided a framework, service model and platform for continual evolution of youth mental health services that has been felt throughout the Edmonton Zone. The service has been required to iterate in order to manage expectations of family and youth as we develop and enhance services, listen to individuals and their families, tackle the practicalities of operating a new type of service (e.g., reception hours, technology infrastructure, utilization of finite space), and a new role of advocating for transforming the status quo for vulnerable youth. Initial informal feedback suggests that accessing care is now easier, provided in a more youth‐friendly environment, provides relevant options and follows a process that enhances self‐determination and a feeling of being in control and collaborating on a youth's health journey.

Because the ACCESS OM site exists in a large urban centre and within a large (province‐wide) health systems delivery organization, there was an immediate need to create operational flexibility in order to achieve the objectives of the project. For example, job descriptions were rewritten to ensure clarity about the expectations of working with young people and related competencies in this area, new postings were developed to attract the appropriate type of applicants, and documentation templates were created to standardize data collection and inform service development.

Given the Edmonton Zone team's focus on youth with complex needs, ACCESS OM has facilitated transition of care to the ACCESS OM clinic after an acute episode of illness or entry to services via urgent access points (such as emergency services), and afforded stronger connections between inpatient and community programs for seamless discharge of those youth requiring inpatient stays. These are new relationships and care pathways that did not exist prior to the opening of the ACCESS OM site but now result in negotiation and collaboration around care transitions between hospital‐ and community‐based teams. In addition to the already‐integrated services (Table 1), our zone Youth Diversion program is also now embedded within the young adult program, allowing connection between youth identified in the criminal justice system that would benefit from treatment within the addiction and mental health system.

## 10 RESEARCH AND EVALUATION

In line with the ACCESS OM research protocol, the Edmonton ACCESS OM site is involved in data collection such as tracking symptom severity, levels of distress, suicidality and goal attainment in order to provide information about the effectiveness of the programming offered. With time, this information will be helpful to determine if the model of care is in fact achieving its goals of strengthening access to services, continuity of care and system response.

At the Edmonton site, the research evaluator is being increasingly integrated into the ACCESS OM clinic process and meets with individuals after their clinical visit, during which time the opportunity to participate in research and evaluation is presented. Peer advisors have worked with the evaluators to modify the request to participate in research and the process of ensuring that consent is as youth‐friendly as possible. One full‐time and two part‐time evaluators meet young people and families in locations of their choice across the city to increase ease and reinforce the value that is placed on their continued participation. Monthly reports from clinic walk‐in data are completed and used to inform development of services, address operational issues and encourage ongoing data quality.

## 11 CHALLENGES AND SUSTAINABILITY

Although some early identification programming has been undertaken, the complexity of opening a single clinic with limited resources in a large city has meant that a more thorough, multi‐pronged outreach effort is required. In this regard, the Edmonton Zone has an addiction and mental health prevention and promotion team whose mandate is to provide public education, student education and general prevention and promotion materials in our community. In the coming year, ACCESS OM Edmonton plans to liaise with this team to design and plan further outreach activities. An associated challenge is that stronger outreach work will likely draw in more youth in need of care, whereas the current clinic site is already outgrowing its space.

Clinically, many of the connections between a help‐seeking youth and an appropriate service provider (e.g., social worker, addiction counsellor, mental health therapist for short‐term therapy, recreation therapy) are made within the first 7 to 10 days. For more intensive care options (e.g., specialty care with a psychologist or specialist psychiatric service), there can be challenges to meeting the target timeline due to limited resources, occasional lack of appropriate care options even within this larger system of care, and continuing gaps in service. And although it is uncommon for youth under 14 to present to the ACCESS OM clinic, well‐developed clinical programming and specialist services for this age range exist elsewhere in Edmonton: in order to conduct initial assessments and ensure connection to the appropriate service, a dedicated child and adolescent psychiatrist is now located on site.

Additional practical challenges include:

1. Difficulties meeting the required target for both recruitment of research participants and data collection for research/evaluation. A number of factors are responsible for this, including initial lack of alignment between data collection and clinical programming and limited staffing by an evaluator. Despite now having two part‐time evaluators, ACCESS OM Edmonton has yet to meet its monthly target for recruitment of participants into research/evaluation.
2. Securing clinic space through a lease and partnership with the YMCA, rather than traditional AHS clinic space
3. Running health informatics infrastructure in a temporary site due to the need for secure connections to transmit health information
4. Changing job descriptions and practice expectations with multiple unions
5. Overall, managing change of such a significant magnitude within a large bureaucratic organization. AHS is transitioning to a new electronic medical record (EMR) provider in late 2019; this may provide opportunities for embedding evaluation tools directly into the new EMR.

Moving forward, the increasing integration of services from a number of sectors/jurisdictions to address the common goal areas of young people and their families accessing services should assist in consistent matching of an individual's needs to available service options. Evaluating this will ensure that services provided can be optimized to meet desired outcomes, and that they are effective and acceptable to young people.

As an outgrowth of ACCESS OM, the AHS‐Edmonton Zone team plans to develop, implement and evaluate a growing number of interventions that will provide more options through which services and treatment plans can be individualized. This has converged around the ACCESS OM framework and facilitated by administrative and psychiatry leadership within the Edmonton Zone Addictions and Mental Health portfolio, who are together working to provide a developmentally relevant continuum of services specifically for this transition‐age group of individuals. Associated with this, the local Mental Health Foundation has been an impactful convener and advocate for commitment to systems change.

Having now firmly established the ACCESS OM site in Edmonton, the service's sustainability is well‐supported by AHS Edmonton Zone, which has allocated operational funds of approximately $1 million/year to increase the capacity of ACCESS OM Edmonton—including case management and psychotherapy interventions, occupational therapy, and partnerships that will further populate the integration of service options.

Finally, a major area of current focus is the scaling up of the single ACCESS OM clinic to a network of integrated youth hubs in the Edmonton Zone. A functional plan has been put forward for a centralized clinical services building and five walk‐in clinic sites to be geographically embedded across Edmonton. This work is developing in consultation with the Mental Health Foundation, with the hope that such an approach will expand across the province of Alberta.

## CONFLICT OF INTEREST

Dr. Malla reports having received honoraria for conference presentations and advisory board participation from Lundbeck and Otsuka, Canada and International in the past three years. None of these have any relation to manuscripts in this supplement.

Dr. Joober reports having received honoraria for conference presentations and advisory board participation from Janssen, Lundbeck, Myelin, Otsuka, Perdue, Pfizer, Shire, and Sunovian; he also received grants from Astra Zeneca, BMS, HLS, Janssen, Lundbeck, and Otsuka; and has royalties from Henry Stewart talks. None of these have any relation to manuscripts in this supplement.

Other authors report no conflicts of interest.
